# Quantifying Crop Rhizosphere Microbiome Ecology: The Next Frontier in Enhancing the Commercial Utility of Agricultural Microbes

**DOI:** 10.1089/ind.2018.29132.pzo

**Published:** 2018-06-01

**Authors:** Paul Zorner, Sean Farmer, Ken Alibek

**Affiliations:** Locus Agricultural Solutions, LLC, Solon, OH

## Introduction

Microorganisms represent the oldest biological forms on the planet and reside in virtually all environments capable of supporting life. These metabolically diverse microscopic organisms are responsible for driving global energy fluxes, including the carbon and nutrient cycles which dramatically affect soil health and nutrient uptake in higher plants, and which support plant growth, health and productivity.^1^ Modern “omics” technologies have been employed to better elucidate the complexities of these microbial-plant relationships. However, these sophisticated methods generate complex data that requires time-consuming interpretation and are too costly to be used “on-farm” by growers as decision making tools to optimize agricultural practices to increase crop productivity. The scientific and academic communities continue to advance our knowledge of complex microbial-plant ecological relationships; but there is an immediate need to develop rapid, low cost diagnostics tools, which growers can use to better apply this growing body of knowledge to their individual farms and successfully increase crop yields. This Commentary briefly reviews current literature documenting the importance of microorganisms to crop productivity and the complex relationship between components of a productive agricultural soil microbiome. We also offer a forward-looking perspective regarding on-farm tools that could enable growers to capitalize in predictable and controllable ways, on this expanding knowledge of how microbial-plant relationships can contribute to improved crop performance to benefit growers.

## Microbial Importance to Productive Agriculture

Microbes are recognized to play crucial roles in agricultural ecosystems.^1–3^ For centuries, farmers have known that plant health and crop yields were directly related to “healthy soils.” During the most recent agricultural revolution, crop yield was the primary focus, and a working definition of healthy soil was generally driven by development of what were considered optimal soil conditions for plant growth such as adequate moisture, organic matter and other physical soil attributes. Synthetic fertilizers were developed as a strategy to apply the macro- and micronutrients needed for optimal crop performance, while chemical pesticides were introduced to control pests and pathogens.^4^ The 20^th^ Century industrialization of agriculture fundamentally changed and intensified the business and agronomic practices of how we grow crops and feed the growing population of the planet. The extensive use of agrochemicals, mono-cropping and tillage has impacted farmlands, and the native productivity of agricultural soils.^5–7^ Global production challenges and economic forces have shifted more attention to the sustainable use of agricultural resources such as soil and water, where it is apparent that intensive agricultural practices if not applied correctly will lead to global soil quality degradation over time. It is in this context that modern biotechnology has demonstrated that the plant rhizosphere microbiome plays a central role as a key mediator between the plant and it's surrounding environment, which in turn impacts crop productivity.^1,3^

Recent studies have illustrated an immense variation in microbial populations residing in diverse geographies and physio-chemical environments. The Earth Microbiome Project findings^8^ at a global level of about 24,000 samples (2,290 plant-associated and 4,279 soil microbe samples) provides the broadest overview, but importantly highlights the limitations of such detailed studies in the absence of comprehensive metadata of the collection sites. Organized metadata provides context and may shed light on important, yet non-obvious patterns. Separate studies focused on soil analysis of 237 sites showed that almost half of the soil bacterial communities were accounted for by 2% of bacterial phylotypes (∼500 phylotypes)^9^ while the parameters (temperature, water/hydration, plants, and soil chemistry) affecting the microbiomes were reviewed from micro- to continent scale.^10^ Together, these studies highlight the opportunity to develop new tools in microbial-crop ecology to quantify the effects of the microbiome in soil environments, and to transition the science from a descriptive to a predictive tool that will enable practical management applications of microbial ecology in agriculture.

The correlation between soil characteristics and microbial populations were further investigated at larger scale in France.^11,12^ Both studies showed that the soil microbiomes were highly dependent on soil properties and land use. More than 2,000 sites were included in the study and soil microbiomes were characterized by 16S rRNA gene sequencing. The parameters investigated also included climatic conditions, geomorphology, land use and space. The soil physico-chemical properties explained most of the variance (18%) and a predictive model for the soil microclimate was developed based on the bacterial species richness.^11^ The total variance explained ranged from 55% to 78%, with microbial biomass and species richness mainly explained by soil pH and texture.^12^

## Crop Rhizosphere Microbiome

Microbes (especially bacteria and fungi) play key roles in plant health both as pathogens, but moreover, also serve as gatekeepers providing the plant with essential nutrients and protection from abiotic stressors.^3^ They facilitate communication between the plant and surrounding soil environment in the rhizosphere. The rhizosphere is the narrow zone of soil surrounding plant roots that is characterized by root exudation and an abundance of microorganisms which can be beneficial or harmful (or no effect) on plant root growth and function. These microbes are saprophytic, phyto-pathogenic or symbiotic bacteria and fungi, including rhizobia forming nodules and arbuscular mycorrhizal fungi.^13^

Taken together the available data suggests that multiple organisms as a group contribute functional genomics and chemistry to clearly interact with each other, plants and the environment in which they all exist to create a productive metagenome which leads to improved crop productivity. The general signal/information flow from plants goes to key members of the microbiome that shape the larger community.^14–16^ Interspecies tree variation was shown to significantly influence the response of the soil microbiome in a controlled greenhouse experiment across several different growth temperatures.^16^ Root length has also been shown to play an important role for the composition and richness of the plant microbiome. By comparing wild and domesticated common bean (*Phaseolus vulgaris*) grown in agricultural soil^17^ showed in this particular study that as the genotype transitioned from wild to domesticated, the relative abundance of *Bacteroidetes* (*Chitinophagaceae* and *Cytophagaceae*) decreased while *Actinobacteria* and *Proteobacteria* (*Nocardioidaceae* and *Rhizobiaceae*) increased. The effect of host-microbe and microbe-microbe interactions on the root and rhizosphere microbiomes in wild and domesticated barley highlighted a similar effect.^18^ Recently, a more detailed understanding of the mechanistic relationship studying annual grass through its developmental stages was proposed.^19^ The study connected comparative genomics and metabolomics to show that specific rhizosphere bacteria are naturally selected depending on the root exudates content of aromatic organic acids (nicotinic, shikimic, salicylic, cinnamic and indole-3-acetic). The potential to predict the recruitment was further highlighted by genome analysis of the representative bacterial isolates.

Less studied parts of the plant microbiome include leaf and fruit tissues. However, evidence of the diversity and function of these microbiomes are also emerging and the comparison to the rhizosphere microbiome has been determined for both model systems^20^ and commercial crops.^21,22^ For example, the ability to culture members of the rhizosphere microbiome from citrus was shown, and known plant growth promoting organisms were isolated, including *Bacillus polymyxa, Azotobacter chroococcum, Bacillus mycoides, Pseudomonas fluorescens*, and *Trichoderma harzianum*.^23^ In citrus greening disease, or huanglongbing (HLB), the pathogen *Candidatus liberibacter* spp., was shown to decrease the abundance of these beneficial members of the plant microbiome.^22,24,25^ Blaustein et al. also analyzed the leaf microbiome and showed that the *Candidatus liberibacter* spp. abundance correlated with the disease progression and decreased the microbiome diversity. The same correlation was found for the root microbiome. Thus, it appears that signaling or the interference of signaling between beneficial microbes, the crop and pathogenic organisms may be a significant factor in the expression and intensity of *Candidatus liberibacter* spp impact on citrus which may in turn provide insight on how to better manage loss of citrus productivity by better understanding these ecological relationships.

Further complex fungal-bacterial associations, quorum sensing between functionally dependent organisms and genomic cross-talk between microbes, fungi and plants have all been recently studied and described.^1,3,26^ Deveau and colleagues describe “fungiphyllic” bacteria that when present confer to the association enhanced functionality to both fungal and bacterial organisms, which in turn confer enhanced plant productivity. Our view is that there is no doubt that there is even more to understand with these remarkably elegant associations. It appears that a productive agricultural environment or ecosystem is modulated by various prokaryotic and eukaryotic organisms each making individually important functional contributions as part of a large metagenome where all members truly communicate with each other to create a productive system that, in the context of agriculture expresses itself as optimized crop productivity. This is not dissimilar to several members of a symphony orchestra each contributing their individual sound and tempo to the orchestrated beauty of a performance, or individual letters in a book contributing to the ultimate meaning of printed verse.

Through this increasingly powerful but still complex science, the agricultural community has come to the realization that soil microbiomes are organized and structured as a response to their surrounding environment such as soil characteristics, which plants are present, what agricultural inputs are applied and tillage practices. Further research will continue to illuminate the importance of these relationships in both the crop rhizosphere and crop endosphere. However, the value of this knowledge will only be as great as the ability of individual growers to apply it to their specific farm, crop, climatic conditions and the multitude of daily, seasonal and annual variables with which growers must deal. The scientific community must not only continue to understand and describe these general functional metagenomic concepts, but also begin to provide growers with on-farm and easy-to-use analytical tools by which growers can measure the key indicators of metagenomic functionality and the related functional “health” of their soils and crop, as a function of the agronomic practices they deploy. This will enable individual growers to measure and adjust their management practices at the field level to optimize functionality and productivity of their specific farm ecosystem.

## Detection, Monitoring and Microbiome Data Synthesis

The ability to study, describe and analyze the plant microbiome has been facilitated by advancements in genome sequencing and the range of ‘omics’ methods. It is not only possible to perform microbial inventories which catalog the abundance of microbial constituents (e.g., 16S rRNA and metagenomics studies), but we can now also assess the metabolic potential of the microbial communities by applying gene-centric metagenome approaches. The Earth Microbiome Project (EMP) focused on 16S rRNA phylogeny and provided a set of protocols that attempted to create datasets comparable across multiple study sites with data generated over time from multiple investigators and sequencing centers.^27,28^ Many studies have highlighted the effect of the methods used on the obtained results from sample collection and storage prior to processing to DNA extraction and potential amplification. DNA extraction methods are very important for soil (especially the initial homogenization step).^29^ Consistent use of methods and standardization is important for spatial and temporal comparisons, both essential factors for development of a better understanding of the plant microbiome and its variability.

A less biased analysis would be based on direct field analysis eliminating the need to preserve and transport the samples to laboratory facilities and carry out the DNA extraction with specialized equipment. The detection technologies for field application has evolved mainly to target applications in human health, but have the potential to be translated to agriculture. Laminar flow assays (LF) are an especially attractive low cost deployment tool. Both immunoassays and nucleic acid detection can be integrated in LF assays.^30–32^ In addition, the combination with nano-sized up-converting phosphor (UCP) reporter particles present an opportunity to further improve the signal-to-noise ratio.^33^ Several studies have highlighted this combination of these technologies for field detection of human pathogens^34,35^ and with the right target, selection based on antibody availability or specific genomics information, can be developed for agricultural applications. Successful deployment would not only enable identification and quantification of both beneficial and pathogenic organisms, but also allow detection of critical functional genes that may be contributed by any microbe, and if not present to provide guidance on what functional characteristics could be delivered as an input to create an optimal and fully functioning metagenome necessary for optimal crop productivity. Thus, on-farm tools would provide the farmer with precise information to implement better real-time management.

## Future Directions Important to Practical Utilization of Microbiome Knowledge

Our ability to describe, and even in some cases elucidate the underlying mechanism for plant microbiome structure at both spatial and temporal variations has been demonstrated. The ability to follow individual microbes and/or the functional genes (chemistry) and how they contribute to the community is also important. The next phase of development for applications in modern agriculture will focus on applying these principles to give the farmers information to inform decisions in real-time. Under development at the author's company, Locus Agricultural Solutions, is a technology platform designed for real-time, multiplexed screening of agricultural micro-organisms using hand-held devices in collaboration with Intelligent Materials Solutions (www.intelligentmaterial.com) which utilize some of the technologies described above by Corstjens; the detection method is based on uniform, nanocrystal phosphors developed by IMS which can accurately detect, quantify and track microbes in the environment. In-field pathogen screening will enable rapid detection and response to nascent spreading disease. In addition, we anticipate the monitoring of soil biome health can be accomplished with real-time assessment of key functional members of the microbial communities residing in crop soils or in the vascular tissues of the crop. The combination of facile pathogen screening with detection of key microbes contributing to optimal rhizosphere function will provide valuable information to growers and farm operators. Industrial biotechnology is rapidly providing new tools and modern genome-methods which will improve agricultural productivity by both maximizing the genetic expression of the plants and optimizing the metagenomic potentials associated with increased crop performance.
